# Predictors of Mortality Subsequent to a Fracture in Diabetes Mellitus Patients

**DOI:** 10.3389/fendo.2015.00046

**Published:** 2015-04-01

**Authors:** Sidse Westberg-Rasmussen, Jakob Starup-Linde, Søren Gregersen, Peter Vestergaard

**Affiliations:** ^1^Department of Endocrinology and Internal Medicine, Aarhus University Hospital, Aarhus, Denmark; ^2^Department of Clinical Medicine, Aalborg University, Aalborg, Denmark; ^3^Department of Endocrinology, Aalborg University, Aalborg, Denmark

**Keywords:** diabetes mellitus, fracture, mortality, statins, antidiabetics

## Abstract

**Background:**

Type-1 and type-2 diabetes mellitus (DM) are associated with an increased fracture risk and possibly an increased risk of death following a fracture.

**Aim:**

To investigate the association between diabetes-related drugs and mortality following a fracture.

**Methods:**

A nested case–control study was conducted. Cases were patients with DM who died following a fracture; controls were DM patients not dying after a fracture. We identified DM patients using the Danish National Hospital Discharge Register (1977–2011) and included information on date of DM diagnosis, date of fracture, and comorbidities. From the Danish Cause of Death Register, the date of death was collected (2008–2011). From the Central Region of Jutland, Denmark, medication use was collected (2008–2011). Analysis was performed by unconditional logistic regression.

**Results:**

Two thousand six hundred twenty one diabetes patients with a fracture following the diabetes diagnosis and with information on medication use were included. Of these, 229 died. In a multivariate analysis, statin use [*n* = 1,106 (42%) statin users, odds ratio (OR) = 0.60, 95% confidence interval, *p* = 0.012] decreased the risk of dying subsequent to a fracture. Male gender (OR = 1.57, *p* = 0.005), increasing age (OR = 1.08, *p* < 0.001), a diagnosis of retinopathy (OR = 2.12, *p* = 0.008), heart failure (OR = 1.68, *p* = 0.004), and use of glucocorticoids (OR = 2.22, *p* = 0.001) were associated with an increased risk of death. None of the antidiabetics: biguanides, glucagon-like receptor agonists, β-cell stimulants, glitazones, and insulin were associated with mortality.

**Conclusion:**

Co-morbidity reflected by late onset complications, heart failure, and glucocorticoid use was associated with an increased risk of mortality subsequent to a fracture. Statin use may reduce mortality subsequent to a fracture in diabetes patients. Clinical trials are needed to determine whether diabetes patients with a fracture should initiate statin treatment.

## Introduction

Diabetes mellitus (DM) is a common disease, with 382 million humans affected worldwide (1). The incidence of type-2 DM (T2DM) is increasing, mirroring the rise in sedentary lifestyle and obesity, while the incidence of type-1 DM (T1DM) has remained relatively constant. Hyperglycemia results in the formation of advanced glycated endproducts (AGEs) and oxidative stress, along with a multitude of other actions. DM is associated with a series of complications (2). The most important complications are macrovascular disease, such as atherosclerosis, and microvascular disease such as retinopathy, nephropathy, and neuropathy. Recently, another complication has been added to the list, namely a negative influence on bone (3, 4). The mechanism behind the impact of diabetes on bone is still widely unknown.

Several studies have shown an association between diabetes and increased risk of fractures (4–6). Furthermore, the use of the WHO fracture risk assessment tool (FRAX) apparently underestimates the risk of fractures in patients with DM (5). Thus, there is a need for alternative tools to predict fracture in diabetic patients. Bone strength depends on both bone quantity and bone quality. As for bone quantity, T2DM patients have higher bone mineral density (BMD), which may be due to obesity, whereas T1DM patients have lower BMD (4). However, the lower BMD in T1DM patients cannot fully explain the higher incidence of fracture among these patients. It seems that DM has an effect on bone quality, on a microarchitectural scale, which leaves bones more porous and prone to fracture.

Risk of mortality and other adverse events due to fractures seem higher in diabetics compared to healthy patients (6–8). Huang et al. (8) showed that not only did DM patients have a significantly higher mortality rate due to fracture but they also had higher readmission rates during the first months following discharge compared to patients without DM. In addition, the DM patients were less likely to have regained walking ability, general health, and physical functioning after a year. Liao et al. (6) found increased mortality rates, higher risk of septicemia, deep wound infection, and urinary tract infection, after a surgically treated fracture compared with patients without DM. This warrants further investigations into the predictors of mortality and adverse effects in diabetics who experience fracture.

The aim of this study is to investigate the association between diabetes related drugs, comorbidities, and mortality due to fracture.

## Materials and Methods

### Design

We conducted a nested case–control study in a cohort of DM patients. Cases were defined as DM patients who died subsequent to fracture in the period from 01-01-1977 to 31-12-2011. Controls were DM patients who had a fracture, but did not die, in the same period. We obtained approval by the Danish Data Protection Agency.

### Diabetes, fracture, and mortality assessment

Using the Danish National Hospital Discharge Register (1977–2011), we included DM patients for whom information on date of DM diagnosis, date of fracture, and comorbidities was available. Only patients for whom the fracture occurred after the DM diagnosis were included. Date of death was collected from the Danish Cause of Death Register. Table 1 gives an overview of the exposure variables applied and corresponding ICD and ATC codes.

**Table 1:** **Overview of included variables and respective codes from ICD or ATC**.

**Diabetes mellitus and fractures (ICD)**
Type-1 diabetes mellitus: DE100, DE101, DE109, 24900, 24909, 24907, 24906
Type 2 diabetes mellitus: DE110, DE111, DE119, 25006, 25007, 25009; fractures: S02.0–S02.9, S07.0–S07.9, S12.0–S12.9, S22.0–S22.9, S32.0–S32.8, S42.0–S42.9, S52.0–S52.9, S62.0–S62.9, S72.0–S72.9, S82.0–S82.9, S92.0–S92.9, 800–808.09, 808.11–808.19, 808.91–816.09, 816.19, 816.99–820.12, 820.18–820.92, 820.98–821.22, 821.28–821.32, 821.38–821.92, 821.98–824.03, 824.08–824.13, 824.18–824.93, 824.98–825.99, 826.01–826.19, 826.99–829.99
**Comorbidities (ICD)**
AMI (DI21, DI22, DI23, 41009, 41099), nephropathy (DE102, DE112, 24902, 25002), neuropathy (DE104, DE114, 24903, 25003), retinopathy (DE103, DE113, 24901, 25001), peripheral artery disease (DE105, DE115, 24904, 24905, 25004, 25005), heart failure (DI50, DI110, DI130, DI132, 42709–42711, 42719, 42899), alcohol (DF10, 303)
**Antidiabetic drugs, antihypertensive drugs, statins (ATC)**
Insulin (A10A), biguanides (A10BA), β-cell stimulating (A10BB), glitazones (A10BG), GLP-1 receptor agonists (A10BX), antihypertensive drugs (C09, C07A, C08, C02AB, C02AC, C02CA, C02DB, C03C, C03AA, C03D), statins (C10AA)
**Antiosteoporotic agents (ATC)**
Hormone replacement therapy (G03), strontium ranelate (M05BX03), teriparatide (H05AA), bisphosphonates (M05BA), denosumab (M05BX)
**Smoking variable (ATC)**
Inhaled steroids (R03BA), vareniclin (N07BA03), nicotine substitution (N07BA01), bupropion (N06AX12), β2-agonist (R03AC), inhaled anticholinergics (R03BB)
**Others (ATC)**
Antipsychotics (N05A), antiepileptics (N03A), antidepressants (N06A), glucocorticoids (H02AB)

### Medication use assessment

From the Central Region of Jutland, Denmark, medication use was collected (2008–2011) by ATC codes (see Table 1). Medication use was defined as having collected a prescription prior to fracture. Only data regarding redeemed drugs on prescription and not over-the-counter drugs were available. In the register, all sales are registered to the individual who redeems the prescription, and therefore the validity of data is high.

Medication types included were anti-smoking medication, antidiabetics; glitazones, biguanides, beta-cell-stimulating medication, glucagon-like peptide (GLP)-1 receptor agonists, and insulin; antiepileptics, antipsycotics, antidepressants, statins, bone active drugs, and antihypertensive medication.

### Proxy variables

A proxy variable for smoking (yes/no) was created by drug usage; yes was defined as use of nicotine substitution, vareniclin, bupropion, inhaled beta-agonists, inhaled anticholinergics, or inhaled corticosteroids. A proxy variable for hypertension (yes/no) was created by drug usage; yes was defined as use of diuretics, beta-blockers, calcium channel antagonists, angiotensin converting enzyme (ACE) inhibitors/AT2 antagonists, antiadrenergic drugs with either peripheral or central effect, renin inhibitors, and hydralazine. A proxy variable for usage of drugs affecting bone (yes/no) was created by drug usage; yes was defined as the use of bisphosphonates, teriparatide, strontium ranelate, denosumab, or hormone replacement therapy (Table 1).

### Statistical analysis

The statistical analyses were conducted using the STATA 8 statistics package. Pharmaceutical use was handled as (yes/no) and biochemical markers were handled as numerical values. An unadjusted case–control analysis and an adjusted case–control analysis were performed using logistic regression. The adjusted analysis was performed to address potential confounding. All results are presented as odds ratios (OR). In the following, the expression risk will be used synonymously with OR.

## Results

### Subject characteristics

Two thousand six hundred twenty-one DM patients with a fracture subsequent to diabetes diagnosis and with information on medication use were included in the study. Of these, 435 had T1DM, 1,155 had T2DM, and the remaining 1,031 had an unspecified diabetes diagnosis. Baseline characteristics are shown in Table 2. When comparing cases with controls, cases were significantly older, with a mean age of 75.7 years (95% confidence interval (CI): 74.2–77.3) compared to 60.6 years (CI: 59.8–61.3) for controls, and had higher prevalence of cardiovascular disease including peripheral artery disease (*p* = 0.004), acute myocardial infarction (*p* < 0.001), and heart failure (*p* < 0.001). Cases had a significantly higher use of glucocorticoids (*p* = 0.013) and lower use of biguanides (*p* = 0.007) and statins (*p* = 0.009) compared to controls. Gender, diabetes duration, previous fractures, smoking, and alcohol were not different between cases and controls.

**Table 2:** **Baseline characteristics of cases and controls**.

Variable	Total (*n* = 2,621)	Cases (*n* = 229)	Controls (*n* = 2,392)	*p*-Value
Age (95% CI)^a^^,^*	61.9 (61.2–62.6)	75.7 (74.2– 77.3)	60.6 (59.8– 61.3)	<0.001
Diabetes duration^a^	7.7 (7.5–7.9)	8.1 (7.5– 8.6)	7.7 (7.4– 7.9)	0.140
Male gender (*n*, %)	1,226 (46.8)	115 (50.2)	1, 111 (46.4)	0.275
Smoking (*n*, %)	239 (9.1)	20 (8.7)	219 (9.2)	0.832
Alcohol (*n*, %)	229 (8.7)	21 (9.2)	208 (8.7)	0.808
Nephropathy^a^	226 (8.6)	28 (12.2)	198 (8.3)	0.079
Neuropathy (*n*, %)	209 (8.0)	19 (8.3)	190 (7.9)	0.850
Retinopathy (*n*, %)^a^	191 (7.3)	22 (9.6)	169 (7.1)	0.210
Peripheral artery disease (*n*, %)^a^^,^*	296 (11.3)	42 (18.3)	254 (10.6)	0.004
Acute myocardial infarction (*n*, %)^a^^,^*	334 (12.7)	55 (24.0)	279 (11.7)	<0.001
Heart failure (*n*, %)^a^^,^*	334 (12.7)	65 (28.4)	269 (11.2)	<0.001
Previous fracture (*n*, %)	872 (33.3)	71 (31.0)	801 (33.5)	0.447
Insulins (*n*, %)	887 (33.8)	66 (28.8)	821 (34.3)	0.093
Biguanides (*n*, %)^a^^,^*	723 (27.6)	47 (20.5)	676 (28.3)	0.007
Glitazones (*n*, %)^a^	12 (0.5)	2 (0.9)	10 (0.4)	0.471
GLP-1 receptor agonists (*n*, %)^a^^,^*	78 (2.2)	1 (0.4)	77 (3.2)	<0.001
β-cell stimulants (*n*, %)	481 (18.4)	47 (20.5)	434 (18.1)	0.374
Glucocorticoids (*n*, %)^a^^,^*	273 (10.4)	37 (16.2)	236 (9.9)	0.013
Antiepileptics (*n*, %)^a^	183 (7.0)	12 (5.2)	171 (7.1)	0.224
Antipsychotics (*n*, %)	120 (4.6)	9 (3.9)	111 (4.6)	0.623
Antidepressants (*n*, %)	570 (21.7)	55 (24.0)	515 (21.5)	0.384
Statins (*n*, %)^a^	1,106 (42.2)	78 (34.1)	1, 028 (43.0)	0.009
Antihypertensives (*n*, %)	1,387 (52.9)	125 (54.6)	1, 262 (52.8)	0.597
Bone active drugs (*n*, %)	219 (8.4)	17 (7.4)	202 (8.4)	0.594

*^a^Bartlett’s test revealed unequal variance, therefore *t*-test for two samples with unequal variance was performed*.

*p < 0.05 between cases and controls.

### Mortality

Of the 2,621 DM patients, 229 died (8.7%). Table 3 shows the crude ORs for the cohort of diabetes patients. When no adjustments for various variables were made, age (OR = 1.07, *p* < 0.001), nephropathy (OR = 1.54, *p* = 0.043), peripheral artery disease (OR = 1.89, *p* = 0.001), acute myocardial infarction (OR = 2.39, *p* < 0.001), heart failure (OR = 3.13, *p* < 0.001), and glucocorticoid use (OR = 1.76, *p* = 0.003) were all associated with an increased risk of death subsequent to a fracture. Biguanides (OR = 0.66, *p* = 0.013), GLP-1 receptor agonists (OR = 0.13, *p* = 0.045), and statins (OR = 0.69, *p* = 0.009) were associated with a lowered risk of death subsequent to a fracture. Gender, diabetes duration, previous fracture, smoking, and alcohol were not associated with the risk of death.

**Table 3:** **Crude odds ratios (OR) for death subsequent to a fracture (*n* = 2,621)**.

Variable (*n*)	OR (95% CI)	*p*-value
Age (per year)*	1.07 (1.06–1.08)	<0.001
Diabetes duration (per year)	1.02 (0.99–1.04)	0.216
Male gender (1,226)	1.16 (0.89–1.53)	0.275
Smoking (239)	0.95 (0.59–1.53)	0.832
Alcohol (229)	1.06 (0.66–1.70)	0.808
Nephropathy* (226)	1.54 (1.01–2.35)	0.043
Neuropathy (209)	1.05 (0.64–1.72)	0.850
Retinopathy (191)	1.40 (0.88–2.23)	0.159
Peripheral artery disease* (296)	1.89 (1.32–2.71)	0.001
Acute myocardial infarction* (334)	2.39 (1.72–3.32)	<0.001
Heart failure* (334)	3.13 (2.29–4.28)	<0.001
Previous fracture (872)	0.89 (0.67–1.20)	0.446
Insulins (887)	0.77 (0.56–1.04)	0.093
Biguanides* (723)	0.66 (0.47–0.91)	0.013
Glitazones (12)	2.10 (0.46–9.64)	0.340
β cell stimulants (481)	1.17 (0.83–1.63)	0.374
GLP-1 receptor agonists* (78)	0.13 (0.02–0.95)	0.045
Glucocorticoids* (273)	1.76 (1.20–2.57)	0.003
Antiepileptics (183)	0.72 (0.39–1.31)	0.281
Antipsychotics (120)	0.84 (0.42–1.68)	0.624
Antidepressants (570)	1.15 (0.84–1.58)	0.384
Statins* (1,106)	0.69 (0.52–0.91)	0.009
Antihypertensives (1,387)	1.07 (0.82–1.41)	0.597
Bone active drugs (219)	0.87 (0.52–1.45)	0.594

*Significant, i.e., *p* < 0.05 between cases and controls.

Table 4 shows the multivariate analysis for the cohort of diabetes patients. In the multivariate analysis, statin use was still found to be associated with a decreased risk of death subsequent to a fracture (OR = 0.60, *p* = 0.012), but the effect of biguanides and GLP-1 receptor agonists were no longer significant. Male gender (OR = 1.57, *p* = 0.005), increasing age (OR = 1.08, *p* < 0.001), a diagnosis of retinopathy (OR = 2.12, *p* = 0.008), heart failure (OR = 1.68, *p* = 0.004), and use of glucocorticoids (OR = 2.22, *p* < 0.001) all increased the risk of death. None of the other antidiabetics: beta-cell stimulants, glitazones, and insulin, were significantly associated with mortality.

**Table 4:** **Multivariate adjusted odds ratios (OR) for death subsequent to a fracture (*n* = 2,621)**.

Variable (*n*)	OR (95% CI)	*p*-value
Age (per year)*	1.08 (1.07–1.10)	<0.001
Diabetes duration (per year)	1.03 (1.00–1.06)	0.088
Male gender* (1,226)	1.57 (1.15–2.15)	0.005
Smoking (239)	0.90 (0.52–1.56)	0.703
Alcohol* (229)	2.29 (1.32–3.97)	0.003
Nephropathy (226)	1.34 (0.83–2.14)	0.228
Neuropathy (209)	0.90 (0.52–1.55)	0.701
Retinopathy* (191)	2.12 (1.22–3.67)	0.008
Peripheral artery disease (296)	1.47 (0.98–2.18)	0.060
Acute myocardial infarction (334)	1.22 (0.84–1.77)	0.307
Heart failure* (334)	1.68 (1.18–2.40)	0.004
Previous fracture (872)	0.87 (0.63–1.21)	0.419
Insulins (887)	1.07 (0.71–1.60)	0.750
Biguanides (723)	1.01 (0.68–1.52)	0.951
Glitazones (12)	3.32 (0.61–18.13)	0.166
β cell stimulants (481)	0.91 (0.60–1.39)	0.676
GLP-1 receptor agonists (78)	0.23 (0.03–1.73)	0.152
Glucocorticoids* (273)	2.22 (1.41–3.49)	0.001
Antiepileptics (183)	0.85 (0.44–1.65)	0.634
Antipsychotics (120)	0.95 (0.44–2.07)	0.904
Antidepressants (570)	1.12 (0.76–1.64)	0.579
Statins* (1,106)	0.60 (0.41–0.90)	0.012
Antihypertensives (1,387)	0.85 (0.54–1.33)	0.472
Bone active drugs (219)	0.83 (0.47–1.47)	0.524

*Significant, i.e., *p* < 0.05.

## Discussion

In the multivariate analysis, male gender, increasing age, a diagnosis of retinopathy, heart failure, and use of glucocorticoids increased risk of death following a fracture. A diagnosis of retinopathy, which is a common complication of diabetes, is associated with an increased risk of all-cause mortality and cardiovascular events in both type-1 and type-2 DM (9). The increased risk of death subsequent to fracture found in patients with retinopathy is therefore to be expected. Heart failure is an indicator of poor general health and is in itself associated with increased mortality, which explains the increased risk of mortality due to fracture; perhaps especially due to the stress induced by a fracture or perhaps surgery for the fracture. Chronic use of glucocorticoids increases the risk of osteoporosis and thereby the risk of fracture. Users of glucocorticoids are often very ill, and glucocorticoid use lowers immune system capacity, which explains why these patients are less resistant to complications from fracture, and therefore more likely to die. Glucocorticoid use may also increase the risk of Addison crisis following a fracture.

The seemingly protective potential of statins in terms of mortality following a fracture in diabetes patients is a novel and very interesting finding. Statins are widely used and well accepted as secondary prevention of cardiovascular events in patients with previous coronary artery disease, in whom they have been shown to lower all-cause mortality (10, 11). Statins lower total cholesterol and low-density-lipoprotein cholesterol in blood (10), which may retard the progression of coronary atheromatous lesions, which subsequently leads to a lowered risk of plaque rupture and thereby coronary events (12). It is debated whether or not statins should be used for primary prevention, e.g., in people with known risk factors but with no history of cardiovascular events. A meta-analysis by Ray et al. (13) found no significant reduction in all-cause mortality when statins were used as primary prevention in intermediate to high-risk individuals without a history of cardiovascular disease. The reduction in mortality due to fracture found in our study may be a reflection of the general all-cause mortality lowering effect of statins in patients with previous cardiovascular disease, or may be due to a direct effect of statins on bone.

In the year 2013, The Danish Diabetes Database (14) found that 35% of T2D in outpatient clinics and 46% of T2D in general practice aged 40 years or more with an LDL cholesterol >2.5 mmol/l did not receive lipid-lowering drugs as suggested by current guidelines. The protective effect of statins present in our study may be due to treatment following guidelines; however, statins may have beneficial effects beside what guidelines suggest, as we are not able to determine the indication of treatment versus non-treatment.

Statin use may also affect the risk of fracture by a direct effect on bone. Many studies have investigated the effect of statins on fracture risk, with some pointing toward a protective effect (15–17) and some pointing toward no effect (18, 19). A meta-analysis from 2007 by Toh et al. (18) found that current evidence does not support an effect of statins in preventing fractures, as there is a lack of association in randomized trials, high heterogeneity among observational studies, and potential residual confounding and publication bias. There has been a lot of debate on the potential “healthy drug user effect,” which suggests that the reason for lower fracture rates among statin users is caused by statin users being more frequent users of medical services and therefore healthier and more aware of healthy behavior. If this was the case, use of other lipid-lowering drugs would be expected to have the same fracture risk-lowering effect. A nation-wide case–control study by Rejnmark et al. (16) showed that non-statin lipid-lowering drugs did not decrease fracture risk, while statins did. This argues against a healthy drug user effect and points toward an actual fracture risk-lowering effect of statins. If statins, as suggested, are protective against fractures only individuals with severely reduced bone status and possibly the most ill have fractures when using statins. This supports the association seen in our study. Thus, statins may both decrease the risk of fracture and decrease the risk of mortality. However, we cannot completely rule out the “healthy drug user effect.”

The suggested biological mechanisms underlying the effect of statins on bone are numerous. Statins competitively inhibit HMG-CoA reductase, which is the rate-limiting enzyme in the pathway that produces cholesterol. One of the other metabolites in this pathway, whose synthesis is also inhibited by statin treatment, is farnesyl pyrophosphate (FPP). FPP inhibits osteoblast differentiation. Thus blockage of FPP synthesis may induce increased osteoblast formation and osteogenesis (20). Another mechanism may be via an effect on the estrogen receptor-alpha (ER-α). Estrogen is an important anabolic factor in bones, and post-menopausal women with lower estrogen levels are more prone to osteoporosis than men and premenopausal women. The effect of estrogen on bone is primarily exerted through the ER-α. Song et al. (21) reported that simvastatin increases the expression of ER-α in murine osteoprecursor cells. This was confirmed in a follow-up study, which also found increased ER-α in ovariectomized rats treated with simvastatin (22). Increased ER-α could lead to an increased sensitivity to the anabolic influence of estrogen on bone. Finally, another possible mechanism may be that statins increase osteoblastic differentiation through enhancement of bone morphogenetic protein-2 (BMP-2) mRNA expression. This effect was seen in murine non-transformed osteoblastic cells and rat bone marrow cells (23). If these biological mechanisms are present in humans, statin use may entail increased fracture healing and thereby increased survival rates, which partly may explain the positive effect of statins on mortality following a fracture. However, further studies are warranted, in order to make conclusions about the above-mentioned pathways in humans.

The strengths of this study are the large group of diabetes patients and the validity of the data registries. This enabled us to make adjustments for numerous variables. We cannot know whether patients actually complied with the medications collected at the pharmacy; however, there is a good chance that patients were motivated to use them, as they collected and paid for them. Our subjects were all Danish citizens. Denmark has a free healthcare system and subsidies are given for prescription drugs. This secures an equal distribution of our subjects among social classes.

This is a retrospective case–control study, which has certain limitations. As different physicians throughout Denmark collected the data used in our study, i.e., diagnosed patients, we rely on other people’s professional, but never the less subjective, opinions. If a physician failed to report symptoms, diagnoses, or outcomes, we have no way of controlling data. Not all diabetes diagnoses were sub-classified as type-1 or type-2 DM. We have no measurements of weight and height and therefore adjustment for BMI was not possible. Furthermore, we did not have actual data on smoking, but used a proxy constructed from diagnoses and drug use known to be associated with smoking. However, the proxy variable for smoking was not associated with mortality, which may question the precision.

## Conclusion

Co-morbidity reflected by late onset complications, heart failure, and glucocorticoid use were associated with an increased risk of mortality subsequent to fracture in diabetes patients. Statin use may reduce mortality subsequent to fracture in diabetes. Clinical trials should decide whether diabetes patients suffering from a fracture should start statin treatment irrespective of current recommendations.

## Author Contributions

PV, SG, JS-L, and SW-R planned and designed the study. JS-L and PV conducted statistics. PV, SG, JS-L, and SW-R interpreted results, drafted, and revised the manuscript. All authors approved the final version.

## Conflict of Interest Statement

The authors declare that the research was conducted in the absence of any commercial or financial relationships that could be construed as a potential conflict of interest.
